# The potential of activator protein 1 (AP-1) in cancer targeted therapy

**DOI:** 10.3389/fimmu.2023.1224892

**Published:** 2023-07-06

**Authors:** Dandan Song, Yan Lian, Lin Zhang

**Affiliations:** ^1^ Clinical Medical Research Center for Women and Children Diseases, Key Laboratory of Birth Defect Prevention and Genetic Medicine of Shandong Health Commission, Shandong Provincial Maternal and Child Health Care Hospital Affiliated to Qingdao University, Jinan, China; ^2^ Department of Obstetrics, Shandong Provincial Maternal and Child Health Care Hospital Affiliated to Qingdao University, Jinan, China

**Keywords:** AP-1, Jun, Fos, cancer, targeted therapy

## Abstract

Activator protein-1 (AP-1) is a transcription factor that consists of a diverse group of members including Jun, Fos, Maf, and ATF. AP-1 involves a number of processes such as proliferation, migration, and invasion in cells. Dysfunctional AP-1 activity is associated with cancer initiation, development, invasion, migration and drug resistance. Therefore, AP-1 is a potential target for cancer targeted therapy. Currently, some small molecule inhibitors targeting AP-1 have been developed and tested, showing some anticancer effects. However, AP-1 is complex and diverse in its structure and function, and different dimers may play different roles in different type of cancers. Therefore, more research is needed to reveal the specific mechanisms of AP-1 in cancer, and how to select appropriate inhibitors and treatment strategies. Ultimately, this review summarizes the potential of combination therapy for cancer.

## 1 Introduction

**Figure 1:**
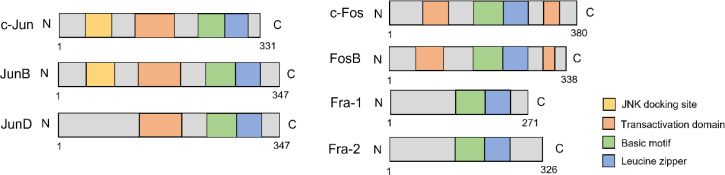
The structure of Jun/Fos family.

The activator protein-1 (AP-1) is a transcription factor discovered in the 1990s (1). It consists of different components, such as the Jun family, Fos family, Jun-dimerizing partners (JDP), musculoaponeurotic fibrosarcoma (Maf) family, and activating transcription factor (ATF) family (2). The Jun and Fos subfamilies are the most predominant and share a conserved bZIP domain that mediates DNA binding (**Figure 1**) (3). While Jun proteins can form both homo- and heterodimers with other proteins, Fos proteins can only heterodimerize with Jun proteins. Furthermore, Fos/Jun heterodimers have greater stability than Jun homodimers (4, 5).

The activated AP-1 can bind to a specific DNA sequence, 5’-TGAG/CTCA-3’, which is located in the promoter or enhancer region, and thereby regulate the transcription of downstream target genes (6). Its DNA-binding activity can be enhanced by 12-O-tetradecanoylphorbol 13-acetate (TPA). Therefore, its DNA binding motif is known as the TPA-response element (TRE) (7). Additionally, AP-1 composed of a Jun/ATF heterodimer has a higher affinity for another DNA sequence, 5’-TGAGCGTCA-3’, which is responsive to cyclic AMP and termed the cyclic AMP responsive element (CRE) (8). Several stimuli can activate AP-1, including extracellular stimulation, such as growth factors, oxidative stress, UV radiation, proinflammatory cytokines, interferon, bacterial, viral infection and intracellular PI3K/Akt and MAPK signaling (6).

Cancer is a complex and heterogeneous disease that involves the dysregulation of multiple cellular processes, such as proliferation, differentiation, migration, and invasion. AP-1 has been described be overexpressed in many tumors, including triple-negative breast cancer (TNBC), colon cancer, classical Hodgkin’s disease, and anaplastic large cell lymphoma (ALCL) (9–11). After activation, AP-1 regulates the expression of downstream genes involved in various aspects of cancer biology, such as cell growth, apoptosis, angiogenesis, invasion, metastasis and drug resistance. For example, to promote angiogenesis and adapt the tumor cells to harsh microenvironments (12). Additionally, it promotes cell proliferation, migration, and invasion after being induced by myeloid differentiation factor 88 (MyD88) in colon cancer (13). A mechanism whereby it impacts cell proliferation, AP-1 can also co-occupy chromatin with YAP/TAZ, nuclear effectors of the Hippo pathway, and regulate downstream genes controlling S-phase entry and mitosis. The association between AP-1 and YAP/TAZ complexes can also promote skin tumorigenesis (14). The removal of the upstream kinase of YAP/TAZ, Lats1/2, led to elevated AP-1 signaling. The activated AP-1 directly interacted with YAP, co-localized in dysplastic lesions, and with this enhanced YAP-induced pancreatic cancer progression (15).

Certain AP-1 members have been described to have roles in tumor cell invasion. Different Jun and Fos members have been described to interact with SMADs responsible for epithelial to mesenchymal transition (EMT) and subsequent invasion of breast cancer. For example, AP-1 members c-Jun and JunB interact with Smad3, and Fra-1 can form complex with Smad2/3 after TGFβ stimulation, while c-Fos is not required for such interaction (16, 17). Club cells in terminal bronchioles and alveolar type 2 pneumocytes (AEC2) cells have been demonstrated to generate lung adenocarcinoma (18). Kadur Lakshminarasimha Murthy et al. found that KRAS mutation can drive club cell and AEC2 cell transformation, during which AP-1 mediated the increase in the epigenome-wide chromatin occupancy. In AEC2 cells, FOSL1-based AP-1 can recruit the BAF (mSWI/SNF) complex to increase chromatin accessibility and control the gene transcription necessary for neoplastic transformation (19, 20). Thus, understanding the structure and function of AP-1, and elucidate its role in cancer is essential for developing novel therapeutic strategies. In this review, we will summary the structure of AP-1, and discuss the potential of AP-1 serving as a target of cancer treatment.

## 2 The Jun family

### 2.1 Structure and regulation

The Jun family has three members, c-Jun (*JUN*), JunB (*JUNB*), and JunD (*JUND*). c-Jun is mapped to chromosome 1p32-31, JunB and JunD are mapped to chromosome 19p13 (21). The Jun family members share common bZIP domain and transactivation domain which is responsible for transcriptional activity and dimerization. c-Jun is intron-less and can be activated at the transcriptional and post-translational levels (22). At the transcriptional level, the c-Jun/AP-1 complex can be activated by the extracellular stimulation and binds to the c-Jun promoter region, thereby forming a positive regulatory loop (23, 24). c-Jun can also be modulated by post-translational modification, including phosphorylation, ubiquitination, and poly(ADP-ribosyl)ation (PARylation) (3, 25). c-Jun can be mainly phosphorylated by c-Jun N-terminal kinases (JNKs), preventing it from degradation (26). Phosphorylation requires the docking site, which mediates the enzyme attraction and phosphorylation. The phosphorylation sites of c-Jun are located at serines 63 and 73 (S63/73) and threonines 91 and 93 (Thr 91/93) (27, 28). It is controversial whether JunB can be phosphorylated by JNK as it lacks these phosphorylation sites. However, Li. et al. revealed that JNK could phosphorylate JunB at Thr102/104 (29). JunD lacks the docking site, but it can be weekly phosphorylated by JNK through homodimer with c-Jun (30). c-Jun can also be phosphorylated by p38 and extracellular-related kinases (ERK1/2, ERK5) (26).

Besides phosphorylation, c-Jun can be modulated by ubiquitin-like protein family members SUMO-1, SUMO-2, and SUMO-3 in Hela cells, which can stop c-Jun entry into the nucleus, inhibit the DNA-binding activity, and negatively regulate its activity (31). Besides, c-Jun can be PARylated by poly(ADP-ribose) polymerase 1 (PARP1) and followed by enhanced DNA-binding activity (32). It has also been demonstrated that c-Jun can be regulated by other mechanisms, such as miRNA and cytoskeleton (2).

### 2.2 Function

c-Jun and JunB are highly expressed in many tumors, such as colon cancer, Hodgkin’s disease, melanoma, and anaplastic large cell lymphoma tissue (9, 33). As an oncogene, c-Jun can mediate migration, invasion and EMT. c-Jun is a primary driver of malignant melanoma tumorigenesis (2). It can also mediate cell apoptosis in response to UV exposure through the p53 pathway (34). Huan et al. revealed that c-Jun interacts with estrogen receptor α (ERα), reprograms ERα chromatin binding, and modulates ER-mediated gene regulation, indicating its potential role in ER-positive breast tumor targeted therapy (35). Besides, c-Jun plays important role in DNA repair response. c-Jun deficient mouse embryonic fibroblasts established a high level of DNA damage (36).

JunB, in some situations, antagonizes c-Jun and inhibits cell proliferation and transformation (3). JunB binds directly to the promoter region of FBXO21, accelerates cartilage degeneration, and further regulates osteoarthritis apoptosis through the JunB-FBXO21-ERK axis (37). Stromal JunB can also serve as a potential suppressor of distant metastasis in breast cancer (38). However, JunB also has oncogenic characteristics, after induced by TGF-β, JunB can further mediate downstream genes involved in tumor invasion and progression (39). c-Jun, JunB, c-Fos, and Fra-1 are all involved in the cell cycle regulation through cyclin A (40).

The Ras signaling pathway is abnormally activated in many human tumors, and c-Jun is required for Ras-related oncogenic transformation (41). Ruiz et al. recently found that c-Jun functioned as a tumor suppressor in the lung adenocarcinoma, while JunD was increased in the absence of c-Jun and was critical for Ras-mediated lung tumorigenesis (42). Jun family tends to have opposite functions in tumor progression. As a proto-oncogene, c-Jun has been described to drive cell proliferation, invasion, and migration, whereas JunD acts opposite to c-Jun, and cannot be induced by TPA (43). In immortalized mouse embryonic fibroblasts (MEFs), c-Jun represses p53 and p21 expression and accelerates cell proliferation, but JunD functions oppositely (44, 45). JunD promoted p53-dependent cell growth in primary fibroblasts, while in the immortalized cell, JunD displayed decreased proliferation which indicates JunD acts in a different way depending on different cell contexts (44).

## 3 The Fos family

### 3.1 Structure and regulation

The Fos family has four members, c-Fos (*FOS*), FosB (*FOSB*), Fra-1 (*FOSL1*), and Fra-2 (*FOSL2*). The Fos family shares common bZIP domain. Besides the N-terminal transactivation domain, c-Fos and FosB have an extra C-terminal transactivation domain responsible for complex assembly and makes the dimer complex more stable (46, 47). Fra-1 and Fra-2 have no transactivation domain, but they can form heterodimers with the Jun family to activate their functions (48, 49). c-Fos and FosB can be induced early in response to extracellular stimulation, but Fra-1 and Fra-2 can react more significantly and last longer, which indicates that the Fra-1 and Fra-2 are essential in maintaining the active status of AP-1 (47, 50).

Like the Jun family, the Fos family can also be regulated at transcriptional and post-translational levels. The c-Fos expression can be regulated by different enhancers, such as cAMP-responsive element (CRE), serum-response element (SRE), and sis-inducible enhancer (SIE) (51–53). The functional TRE, SRE, and activating transcription factor (ATF) sites were found in the Fra-1 or Fra-2 promoter/enhancer region, indicating Fra-1 and Fra-2 can be auto-regulated by AP-1 (54, 55). This can be a reason for the delay of Fra-1 and Fra-2 expression in response to the extracellular stimulation.

The activity and degradation of the Fos family are mainly mediated by phosphorylation. The c-Fos can be phosphorylated by ERK and its substrates at Thr325, Thr332, Thr232, Ser374, and Ser362 (56); it can also be phosphorylated by p38 at Thr232, Thr325, Thr331, and Ser374 (57). Fra-1 can be phosphorylated at Ser252 and Ser265 (56). Dimerization with c-Jun increases c-Fos nuclear retention inhibits nuclear exit, and enhances its transcriptional activity, while JunB and JunD are less efficient at inhibiting c-Fos shuttling (58). c-Fos can also be PARylated by PARP1, but as it has to form heterodimers with the Jun family, its DNA-binding activity is not enhanced by PARP1 (32). Like c-Jun, c-Fos can also be SUMOylated by SUMO-1, SUMO-2, and SUMO-3, consequently negatively regulate its transcriptional activity (31).

### 3.2 Function

As described before, c-Fos and Fra-1 were involved in cell cycle regulation through cyclin A. Brown et al. also elucidated that c-Fos and FosB served as a direct or indirect transcriptional regulator of cyclin D1 (59). They also found that c-Fos and FosB double knockout (KO) mice had similar phenotypes to c-Fos single KO mice, such as osteopetrosis and failure of tooth eruption, but double KO mice were 30% smaller (59). Fra-1 KO mice resulted in embryonic death, and Fra-2 KO leads to death after birth (60, 61), indicating that Fra-1 and Fra-2 play an essential role in embryonic development.

The Fos family also plays an important role in tumor progression. Fra-1 was positively correlated with cancer malignancy, proliferation, and invasion (4, 62). Evidence shows that Fra-1 can promote colon cancer cell motility and invasion without affecting proliferation (63). Fra-1 can also active EMT, a hallmark of reduced cell-cell adhesion and increased cell motility (11, 64, 65). The EMT relates to organogenesis, morphogenesis, homeostasis, and tumor initiation and is responsible for chemo- and immunotherapy resistance (66). Data indicated that Fra-1 could bind directly to several EMT activators, including SNAI2 and ZEB1 in colon cancer and ZEB2 in TNBC cells, and mediate downstream gene expression (11, 67, 68). The Weinberg laboratory found that EMT transcriptional factors Twist and Snail can bind to the first intron and transcriptional start site region of *FOSL1* and activate Fra-1 in immortalized human mammary epithelial cells (69). Thus, Fra-1/EMT transcription factors form a positive loop to highlight its role in tumor progression. It is interesting to note that during EMT, Fra-1 was found to replace c-Fos to form a heterodimer with c-Jun (69). At the gene level, *FOSL1* predicts poor distant metastasis-free survival (DMFS), while *FOS* and *FOSB* indicate better survival (69). Fra-1 is undetectable in most FosB positive breast cancer cells, but it is expressed in FosB negative cell lines (70). Above all, various studies indicated the potential of Fra-1 in cancer targeted therapy.

## 4 AP-1 inhibitors and its potential in combination therapy

### 4.1 AP-1 inhibitors and clinical trials

#### 4.1.1 T-5224

**Figure 2:**
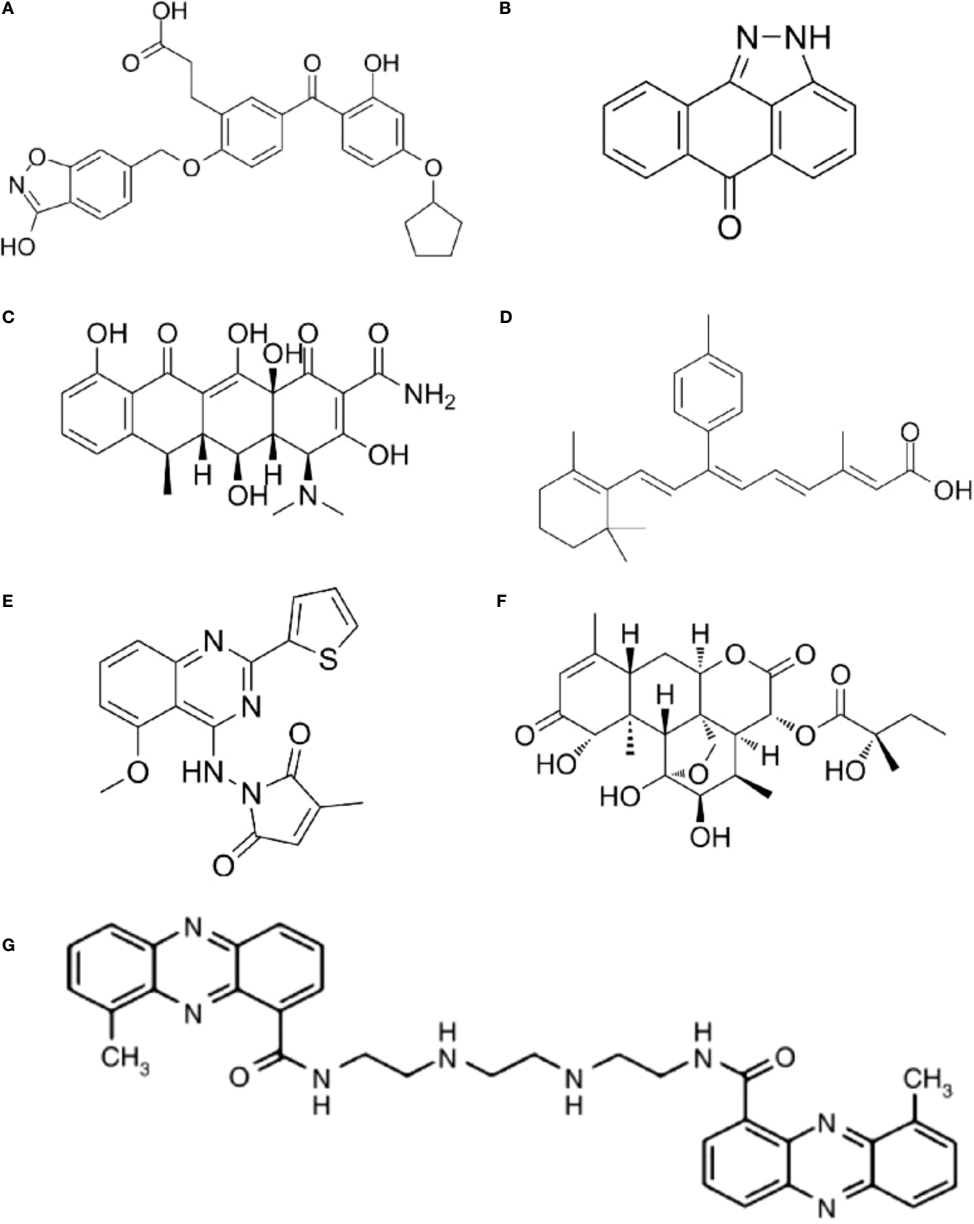
The structure of AP-1 inhibitors. **(A)** T-5224: Reuse from MedChemExpress, CAT: HY-12270, CAS NO.: 530141-72-1. **(B)** SP600125: Reuse from MedChemExpress, CAT: HY-12041, CAS NO.: 129-56-6. **(C)** Doxycycline: Reuse from MedChemExpress, CAT: HY-N0565, CAS NO.: 5564-25-0. **(D)** SR11302: Reuse from MedChemExpress, CAT: HY-15870, CAS NO.: 160162-42-5. **(E)** SPC839: Reuse from MedChemExpress, CAT: HY-10072, CAS NO.: 219773-55-4. **(F)** (+)-Glaucarubinone: Reuse from MedChemExpress, CAT: HY-N10926, CAS NO.: 11259-86-5. **(G)** XR5944: Reuse from Br J Cancer 2007, 97(7): 844-850. Verborg W et al., First-into-man phase I and pharmacokinetic study of XR5944.14, a novel agent with a unique mechanism of action (71). Copyright 2007 is provided under the terms of the Creative Commons CC BY License (http://creativecommons.org/licenses/by/4.0).

As different AP-1 components have reverse functions in cancer development, therapeutic strategies targeting AP-1 should be specifically designed and carefully explored in clinical usage. Yukihiko et al. designed a small molecule c-Fos/AP-1 inhibitor (T-5224) using a three-dimensional pharmacophore modeling based on the crystal structure of the AP-1-DNA complex (**Figure 2A**) (72). It can specifically inhibit AP-1 binding to the DNA, and it has been proven to resolve arthritis in a preclinical model (72). To be noted the daily dose of the T-5224 could be administered up to 150mg/kg in rats and ≥ 750 mg/kg in cynomolgus monkeys for 1 month without observed adverse effects. Evidence showed that T-5224 inhibited matrix metalloproteinase expression in human articular chondrocytes and, hereby, prevented cartilage destruction in an osteoarthritis-induced mouse model (73). Daisuke et al. proved that T-5224 can prevent invasion and migration in head and neck squamous cell carcinoma cells and oral administration of T-5224 inhibit lymph node metastasis in head and neck cancer in an orthotropic tumor implantation mouse model without affecting the tumor growth (74). A daily dose of 150 mg/kg has been confirmed to be safe in rodents, and a lower dose can be used for some inflammatory diseases (74). The efficiency of T-5224 *in vivo* is about 10 times more effective than *in vitro*. The high efficiency may be due to its crosstalk with IL-1, IL-6, TNF-α, MMPs, etc. (72, 74). Also, the crosstalk might explain why there are few adverse effects with the AP-1 inhibition.

#### 4.1.2 SP600125 and doxycycline

JNK inhibitor, SP600125 (**Figure 2B**), which can block AP-1 phosphorylation and stop its activation, showed a protective role in atherosclerosis initiation in apolipoprotein E-deficient mice (75). As *in vitro* and animal studies revealed AP-1 had a critical role in the initiation and progression of vascular dysfunction and atherogenesis (75, 76), hence, Meijer et al. tested whether doxycycline (**Figure 2C**), which had a direct inhibitory effect on JNK1/2 can improve vascular function in a double-blind placebo-controlled cross-over trial (Dutch Trial Registry NTR1389) (77). Results indicated that minimal activation of AP-1 was found in non-progressive and progressive phases of atherosclerosis respectively, and no significant difference was found between progressive and vulnerable lesions. Thus, the clinical trial didn’t confirm AP-1’s role as a therapeutic target for human atherosclerotic (77). It seems the clinical trial had reverse results with the animal studies, in which AP-1 inhibition in hypercholesterolemia mice finally prevent atherosclerosis. However, atherosclerotic in human were in advanced stage, but AP-1 activation occurred much earlier. Also, they found a clear protective effect on vascular inflammation in human abdominal aortic aneurysm (77). The different results might be because AP-1 has a prominent role in aneurysmal disease but has less function in advanced atherosclerotic disease (78).

#### 4.1.3 MLN944 (XR5944) and other inhibitors

The MLN944 is a novel cytotoxic drug with anti-tumor activity against human and murine tumor models both *in vivo* and *in vitro* (**Figure 2G**) (79). The MLN944 can block c-Jun binding to the AP-1 site hence block AP-1 transcriptional activity. Preclinical studies showed MLN944 delayed tumour growth in the HT29 human colon carcinoma. Further clinical trail has been conducted with MLN944 for treatment with patients with advanced tumours to determine the dose-limiting toxicity. While the lack of correlation between toxicity and pharmacokinetics values made it difficult to continue for phase II clinical trail (71).

The SR11302 inhibit Fra-1/AP-1 binding to TRE site showed significantly suppression in tumor growth and lymph node metastasis of head and neck squamous cell carcinoma (HNSCC) (**Figure 2D**) (80). The SPC839 is a dual inhibitor of AP-1 and NF-κB, showed good inhibitory activity against nitric oxide, TNF-α and FLT3, which is a potential target for acute myeloid leukemia (AML) (**Figure 2E**) (81, 82). The glaucarubinone can block AP-1 promoter result to inhibiting cell growth (**Figure 2F**) (83), but it was not specific to AP-1. Even there are various AP-1 inhibitors used for research, but with low-specificty and unpromising *in vivo* results, few applied in clinical trails (**Table 1**).

**Table 1:** The inhibitors of AP-1.

Inhibitor	Target	Strategies	Application	Reference
T-5224	c-Fos/AP-1	Inhibition of protein-DNA binding	Arthritis	(72)
SP600125	JNK1/2	Inhibition of AP-1 activation	Atherosclerosis	(75, 84)
Doxycycline	JNK1/2	Inhibition of AP-1 activation	Atherosclerosis	(77, 85)
MLN944 (XR5944)	c-Jun/AP-1	Inhibition of protein-DNA binding	Cancers	(71, 79)
SR11302	Fra-1/AP-1	Inhibition of protein-DNA binding	Head and neck squamous cell carcinoma	(80)
SPC839	AP-1/NF-κB	Inhibition of transcriptional activity	AML	(81)
(+)-Glaucarubinone	AP-1	Inhibition of AP-1 transcription		(83)

### 4.2 Potential for modulating AP-1 to enhance targeted therapy

AP-1 is active/overexpressed in many tumors and has multi-roles in different cancer progressions. Various studies have revealed its therapeutic potential in cancer treatment, while, AP-1 inhibition independently got limited effect in clinical trials. Meanwhile, AP-1 can drive resistance to cancer treatment. For example, Rampioni et al. revealed the resistance effect of miR-301a/Fra-2/GLIPR1 axis in lung cancer cisplatin treatment (86). Hence, AP-1 targeted therapy has been explored in various combination therapy for cancer treatment.

#### 4.2.1 AP-1 in immune therapy

Immunotherapy or immune checkpoint blockade (ICB) therapy has been proven efficient in metastatic cancers, such as lung cancer, melanoma, and breast cancer (87–89), leading to improved overall survival. Immune checkpoints (e.g., PD-1/PD-L1 and CTLA-4) function in maintaining self-tolerance and restricting immune response. They are frequently exploited by the tumor cells to escape the immune system, which results in immune escape (90). The ICB is beneficial only for a limited fraction of cancer patients. Factors that affect checkpoint expression have been regarded suppressing the ICB efficiency. Among them, AP-1 has been described as an important factor regulating checkpoint expression. Researches revealed c-Fos bound to the promoter region of PD-1 and regulate its expression; Fra-1 mediated PD-L1 expression; c-Jun and JunB can also bound to the enhancer region of PD-L1 (91–93). Thus, AP-1 targeted inhibition can reduce immune checkpoint expression and improve the sensitivity of patients’ response to ICB therapy.

Besides, AP-1 was described to regulate the immune system during cancer development (94). T cell anergy states the unresponsive status of T cells, and T cell exhaustion refers to the states of CD8^+^ T cells, which respond poorly during chronic infection or cancer (95, 96). Evidence suggested that AP-1 cooperated with NFAT regulated gene expression after immune response, hence lack of AP-1 led to blockage of T cell activation and eventually resulted in T cell energy (97, 98). In addition, exhausted cells exhibit low expression of AP-1 factors (Fos, FosB, and JunB) (99). JunB plays a vital role in T cell differentiation and proliferation in multi-ways: it is responsible for IL-2Rα expression in cooperation with other AP-1 components; it contributes to IL-2 production in conventional T cells; forms heterodimer with BATF to regulate the expression of Th-17-related factors (100). JunB can also bind directly to the promoter region of IL-4, therefore promoting its expression during the differentiation of T helper 2 (Th2) cells (45).

c-Jun can enhance T cell functional capacity and promotes its anti-tumor potency. T cell exhaustion has been regarded as a cause of Chimeric antigen receptor (CAR)-T cell dysfunction, so editing exhaustion resistant CAR-T cells can enhance its anti-tumor activity and improve clinical outcomes (101, 102). c-Jun overexpression can exhibit CAR-T cell exhaustion resistance, improve expansion potential, reduce terminal differentiation, and improve its anti-tumor potency in *in vivo* models (103). c-Jun overexpression reduced the T cell exhaustion associated genes and increased memory genes (103) Lynn. et al. described c-Jun enhanced CAR-T cell anti-tumor activity in a Nalm6-GD2^+^ leukemia model and dramatically increased T cell expansion, preventing 143B osteosarcoma tumor growth *in vivo* (103).

Therefore, AP-1 can mediate ICB expression to block immune response, and on the other hand, AP-1 can drive immune system activation. Different AP-1 components in different cells may play a reverse effect in regulating the immune response. Specific AP-1 members in target tissues should be further explored to enhance ICB.

#### 4.2.2 AP-1 in PARP1 inhibitor therapy

Poly(ADP-ribose)polymerase 1 (PARP1) can transfer ADP-ribose units from nicotinamide adenine dinucleotide (NAD^+^) to target proteins, such as histones, DNA polymerase, DNA ligases, and itself (104, 105). This process is called poly(ADP-ribosyl)ation (PARylation). PARP1 functions in single-strand DNA break (SSB) repair through base excision repair (BER) (106). When the SSB repair is blocked by PARP1 inhibitor, SSB can accumulate to double-strand break (DSB), which can be repaired by BRCA-mediated homologous recombination (107). This is the basis of PARP1 inhibitor target therapy for BRCA-mutated breast cancer and ovarian cancer.

PARP1 inhibitor has been applied in the clinic to treat BRCA1/2 mutated breast and ovarian cancer. Our group applied multi-omics approach revealed PARP1 can PARylate Fra-1 and repress its expression in TNBC cells; PARP1 inhibitors can increase Fra-1 expression, and the increased Fra-1 results in the resistance to PARP1 inhibitors (92). Evidence has also shown that PARP1 PARylates c-Jun/c-Fos and promotes AP-1 phosphorylation and DNA-binding activity (32, 108, 109). As PARP1 inhibition can increase Fra-1 expression without affecting c-Jun expression (92), AP-1 (Fra-1/c-Jun) inhibition combined with PARP1 inhibition therapy may serve as a new joint therapy for breast and ovarian cancer patients. As AP-1 is overexpressed in TNBC, the new joint therapy may be applied in TNBC patients.

#### 4.2.3 AP-1 in CDK4/6 inhibitor therapy

Cyclin-dependent kinases 4 and 6 (CDK4/6) mediate the cellular cell cycle by transitioning the G1 to S phase. The CDK4/6 inhibitors, as a result of this, induce G1 cell cycle arrest in tumor cells (110). They are prescribed routinely in clinical to treat estrogen receptor-positive breast cancer. Trials that apply it against other cancer types (e.g., Human epidermal growth factor receptor 2 positive breast cancer, triple-negative breast cancer) are ongoing (111). Watt et al. revealed that CDK4/6 inhibitor increased AP-1 components (e.g., c-Jun, JunB, Fra-2) expression, driving its transcriptional activity (112). Therefore, AP-1 may mediate the resistance of the CDK4/6 inhibitor therapy. Thus, AP-1 blockage may sensitize the CDK4/6 inhibitor therapy and improve patients’ outcomes.

#### 4.2.4 AP-1 in HDAC targeted therapy

The histone modulation, including acetylation, and methylation, alters the structural interaction between the histone proteins and DNA and modulates the DNA transcription and protein expression (113). Histone acetylation is controlled by histone acetyltransferases and deacetylases, the aberrant histone acetylation is associated with many tumors (114). The histone deacetylase (HDAC) inhibitors have been used in clinical for various cancers, including cutaneous T cell lymphoma (113), breast (115), non-Hodgkin’s lymphoma, and mantle cell lymphoma (114, 116). Yuan et al. revealed that HDAC inhibition could promote c-Fos expression without affecting c-Jun expression. The increased c-Fos activated AP-1 formation and mediated the resistance of HDAC targeted therapy (117). Thus, the c-Fos/AP-1 may be the side effect of HDAC targeted therapy, and c-Fos/AP-1 inhibition may improve the efficiency of HDAC targeted therapy.

#### 4.2.5 AP-1 in NF-κB targeted therapy

The AP-1 and NF-κB dual inhibitor SP100030 is one of the first reported small molecules to inhibit gene expression induced by stimuli (118). It can recover muscle weight by increasing MyoD gene expression in the cachectic tumor-bearing rat (119). Suto et al. constructed an adenovirus-expressing TAM67, a dominant-negative mutant of c-Jun (120). It has no transactivation domain and can inhibit the endogenous AP-1. The TAM67 has been shown to reduce the tumor volume in the xenograft mice model, indicating that TAM67 may be a new cancer treatment strategy.

### 4.2.6 AP-1 in EGFR targeted therapy

EGFR targeted therapy is mainly used for lung cancer especially non-small cell lung cancer (NSCLC) that has EGFR mutations or amplification. While it has limitations, such as toxicity and resistance (121). c-Jun was described to mediate EGFR targeted therapy (gefitinib) resistance in NSCLC (122). The, gefitinib-resistant cells displayed high expression of c-Jun and c-Jun interacting proteins. The c-Jun also showed higher occupancy at the JUN transcription start site, which suggested a positive feedback loop maintains a high basal level of c-Jun. Thus, c-Jun inhibition may sensitize EGFR targeted therapy in NSCLC.

## 5 Future perspective

AP-1 is involved in various aspects of tumorigenesis. It has critical role in driving cancer progression by controlling cellular processes such as proliferation, invasion, EMT, metastasis and therapeutic resistance. Despite the demonstrated potential of AP-1 as a target in cancer therapy, the precise functions and mechanisms of AP-1 remain incompletely understood. To fully harness the therapeutic potential of AP-1, it is essential to expand our understanding of its roles and interactions in gene expression across different cancer types and cellular contexts.

Even AP-1 targeted therapy may be a potential choice for cancer, there are still challenges and limitations that need to be addressed. First, specific AP-1 components are known to harbor unique functional roles that need further investigation. Targeting the individual subunits of AP-1 may result in more specific and effective inhibition of its oncogenic functions. However, the development of specific AP-1 inhibitors that can target individual subunits is a prerequisite. Unfortunately, the current lack of such inhibitors impedes the development of targeted AP-1 therapies. With a more comprehensive understanding of the regulatory mechanisms and functional roles of AP-1, we may be able to design better targeted therapies that can prevent or inhibit cancer progression in specific cellular contexts, leading to improved clinical outcomes for cancer patients.

Also, as an indispensable transcriptional factor, AP-1 regulate gene expression in response to various stimuli. Unspecific inhibition of AP-1 may induce unpredictable side effect. Future research should also focus on how to deliver inhibitors specific to target site. Above all, targeting AP-1 independently for cancer therapy is a challenging strategy, further studies should also focus on the combination therapies which could be a more promising strategy for cancer therapy.

## Author contributions

DS drafted manuscript. YL and LZ edited and revised manuscript; all authors approved final version of manuscript. All authors have read and agreed to the published version of the manuscript.
